# The Microstructural Plasticity of the Arcuate Fasciculus Undergirds Improved Speech in Noise Perception in Musicians

**DOI:** 10.1093/cercor/bhab063

**Published:** 2021-05-26

**Authors:** Xiaonan Li, Robert J Zatorre, Yi Du

**Affiliations:** CAS Key Laboratory of Behavioral Science, Institute of Psychology, Chinese Academy of Sciences, Beijing 100101, China; Department of Psychology, University of Chinese Academy of Sciences, Beijing 100049, China; Montréal Neurological Institute, McGill University, Montréal, QC H3A 2B4, Canada; International Laboratory for Brain, Music, and Sound Research (BRAMS), Montréal, QC H3A 2B4, Canada; Centre for Research on Brain, Language and Music (CRBLM), Montreal, QC H3A 2B4, Canada; CAS Key Laboratory of Behavioral Science, Institute of Psychology, Chinese Academy of Sciences, Beijing 100101, China; CAS Center for Excellence in Brain Science and Intelligence Technology, Shanghai 200031, China; Department of Psychology, University of Chinese Academy of Sciences, Beijing 100049, China; Chinese Institute for Brain Research, Beijing 102206, China

**Keywords:** arcuate fasciculus, mediation analysis, musical training, speech-in-noise perception

## Abstract

Musical training is thought to be related to improved language skills, for example, understanding speech in background noise. Although studies have found that musicians and nonmusicians differed in morphology of bilateral arcuate fasciculus (AF), none has associated such white matter features with speech-in-noise (SIN) perception. Here, we tested both SIN and the diffusivity of bilateral AF segments in musicians and nonmusicians using diffusion tensor imaging. Compared with nonmusicians, musicians had higher fractional anisotropy (FA) in the right direct AF and lower radial diffusivity in the left anterior AF, which correlated with SIN performance. The FA-based laterality index showed stronger right lateralization of the direct AF and stronger left lateralization of the posterior AF in musicians than nonmusicians, with the posterior AF laterality predicting SIN accuracy. Furthermore, hemodynamic activity in right superior temporal gyrus obtained during a SIN task played a full mediation role in explaining the contribution of the right direct AF diffusivity on SIN performance, which therefore links training-related white matter plasticity, brain hemodynamics, and speech perception ability. Our findings provide direct evidence that differential microstructural plasticity of bilateral AF segments may serve as a neural foundation of the cross-domain transfer effect of musical experience to speech perception amid competing noise.

## Introduction

There is a great deal of evidence that musical training experience has a pervasive positive effect on auditory cognitive functions (e.g., working memory, language skills) and results in wide-spread structural and functional changes in the human brain (Kraus and Chandrasekaran 2010; Herholz and Zatorre 2012). However, surprisingly, little is known about the relationship between behavioral improvements and specific neurobiological changes. Speech perception in noisy environments is one of the critical abilities that is frequently demonstrated to be improved in musicians (Coffey et al. 2017a). Since speech and music are 2 communication systems fundamental for human social interaction, musicians’ advantage in speech-in-noise (SIN) perception represents a valuable model for studying the cross-domain transfer effect of musical experience. To date, no study has investigated the brain structural correlates of strengthened SIN perception in musicians. The answer to this question is important for understanding the shared neural resources underlying musical and speech processing, and the application of more targeted musical therapy to restore speech functions in neurological and developmental disorders or in aging population (Zendel et al. 2019).

SIN perception, or the “cocktail party phenomenon,” requires skilled perceptual and cognitive processes (segmenting, grouping, representing, and storing target acoustic signals) in order to pick up meaningful units (Parbery-Clark et al. 2009). The musician advantage in SIN perception has been associated with more faithful spectral and temporal encoding of speech sounds along the auditory pathway (Parbery-Clark et al. 2012; Kühnis et al. 2013; Coffey et al. 2017b). In addition, enhancement of higher-level cognitive processes, such as auditory attention and working memory, has been correlated with improved SIN perception in musicians (Strait and Kraus 2011; Kraus et al. 2012; Puschmann et al. 2019; Yoo and Bidelman 2019; Zhang et al. 2021). Musicians also exhibit more robust specificity of phoneme representations in frontal articulatory system and auditory regions as well as stronger intra- and interhemispherical functional connectivity between those regions than nonmusicians in a SIN task (Du and Zatorre 2017). According to the analysis-by-synthesis model, feedforward articulatory predictions are generated to assist the perception of acoustic patterns under noisy and uncertain listening contexts (Poeppel and Monahan 2011). Since sensorimotor interplay is ubiquitous in playing music that results in pervasive structural and functional plasticity in the overlapped sensorimotor network implicated in both the production and perception of music and speech (Hickok and Poeppel 2007; Zatorre et al. 2007; Bailey et al. 2014), it is hypothesized that musicians would benefit from strengthened sensorimotor integration function when processing speech, particularly in challenging listening environments. Indeed, greater task-related (Du and Zatorre 2017) and resting (Luo et al. 2012; Palomar-Garcia et al. 2017; Zamorano et al. 2017) functional connectivity in auditory–motor networks have been found in musicians than nonmusicians. Compared with nonmusicians, musicians also exhibited stronger structural connectivity in sensorimotor circuits including the arcuate fasciculus/superior longitudinal fasciculus (AF/SLF) (Oechslin et al. 2010; Halwani et al. 2011; Giacosa et al. 2016).

The AF/SLF has been proposed as the anatomical basis of the auditory “dorsal stream” for mapping speech phonological information onto articulatory motor representations (Hickok and Poeppel 2007; Saur et al. 2008). Note that, historically, the AF and SLF have been viewed as synonymous fiber pathways, but recently, they are identified as partially overlapped tracts with differential termination regions (Frey et al. 2008; Gierhan 2013; Martino et al. 2013; Chang et al. 2015). According to the widely used segmentation approach by Catani et al. (2005), the AF, including the classical AF and most portions of SLF, has 3 segments: a long direct frontotemporal segment, an anterior indirect frontoparietal segment (overlapping with SLF-II and SLF-III), and a posterior indirect temporoparietal segment (belonging to the temporal part of SLF, SLF-tp) (Wang et al. 2016). Nonetheless, the exact functional specialization of the 3 AF segments is far from definite.

Based on lesion studies and brain electrical stimulation, the direct AF has been implicated in syntactic and phonological processing, the anterior AF in articulation, and the posterior AF in phonological processing (Duffau 2008; Gierhan 2013; Chang et al. 2015). Using diffusion tensor imaging (DTI), it was found that the microstructure of the left direct AF was associated with auditory word learning ability (López-Barroso et al. 2013) and phoneme awareness (Vandermosten et al. 2012), the microstructure of the left anterior AF correlated with speech imitation ability (Vaquero et al. 2017), whereas abnormal microstructure of the left posterior AF was related to impaired phonological working memory in patients with reading disability and autism (Lu et al. 2016). Directly related to the present study, the fractional anisotropy (FA) of the left posterior AF significantly correlated with performance of perceiving speech sentences in noise in 20 dyslexic and 20 control participants when group type, IQ, and quality index of DTI acquisition were controlled (Vandermosten et al. 2012), whereas the mean diffusivity of the left direct AF mediated the aging effect on syllable in noise discrimination sensitivity (Tremblay et al. 2019).

However, the white matter substrates supporting SIN perception in musicians have not been studied. In contrast to the relevance of speech and language function with AF microstructure in the left hemisphere, the right AF has been implicated in music processing. For instance, the structure of the right AF predicted melody and rhythm learning speed (Vaquero et al. 2018) and pitch-related musical grammar learning performance (Loui et al. 2011) in nonmusicians, and abnormal right AF was identified in congenital amusia (Peretz 2016; Chen et al. 2018) and acquired amusia (Sihvonen et al. 2019). Moreover, musical training has been associated with increased tract volume in the right AF and higher FA value in the left AF (Halwani et al. 2011), enlarged F1 (a directional diffusivity measure) value of the right AF/SLF (Giacosa et al. 2016), as well as enhanced volume in the right direct AF that reduced the normative leftward asymmetry of the direct AF (Vaquero et al. 2020). In general, it remains unclear how musical experience modulates the microstructure of bilateral AF segments, and more importantly, whether those features are related to superior SIN perception ability in musicians.

In the present DTI study, we aimed at investigating whether and how long-term musical training modulates the white matter diffusivity of bilateral AF segments and its contribution to SIN perception ability. The corticospinal tract (CST), which is part of the sensorimotor system and connects cortical motor regions with brainstem, is often modulated by musical experience (Giacosa et al. 2016; Imfeld et al. 2009), but should not be related to audition, and thus served as a control tract in the current study. The diffusivity values and lateralization pattern of each tract were compared between a group of musicians and a group of nonmusicians who had participated in our previous functional magnetic resonance imaging (fMRI) study (Du and Zatorre 2017); and more importantly, these values were correlated with SIN perception performance. As shown above, the right AF is consistently reported as a key tract with musical experience-dependent plasticity. We hypothesized that compared with nonmusicians, musicians might exhibit changed diffusivity in the right direct AF and altered AF asymmetry that would scale with individual’s SIN perception ability. Moreover, as shown in our previous fMRI study, the blood oxygenation level-dependent (BOLD) activity in auditory areas of the right superior temporal gyrus (STG) predicted SIN perception accuracy in musicians (Du and Zatorre 2017). Since the right STG is a terminal region of the right direct AF, a mediation analysis was further carried out to test our assumption that the diffusivity of the right direct AF leveraged on SIN perception performance via the BOLD activity in the right STG as a mediator. The rationale for the model selection is that the long-term structural basis is more likely to influence the immediate hemodynamic activity in the target region, which in turn contributes to behavioral performance (structure $\rightarrow$ function $\rightarrow$ behavior), than the other way around (function $\rightarrow$ structure $\rightarrow$ behavior). By doing so, it could link the white matter microstructural reorganization and hemodynamic functional changes in the auditory–motor network with SIN behavior, which would yield new insights into the neural foundations of speech perception advantage associated with musical expertise.

## Materials and Methods

### Participants

Participants include 15 musicians and 15 nonmusicians from a previous fMRI study (for details, see Du and Zatorre 2017). All of them were young healthy right-handed native English speakers with normal hearing and gave written informed consent approved by the McGill University Health Centre Research Ethics Board. One musician and 1 nonmusician were excluded from analyses because of missing diffusion tensor images. The 2 groups were matched for gender (7 females in each group), age (musicians: 20.93 ± 2.06; nonmusicians: 21.86 ± 4.40; *t*_26_ = −0.72, *P* = 0.48), postsecondary education years (musicians: 13.64 ± 3.57; nonmusicians: 14.36 ± 4.22; *t*_26_ = 0.48, *P* = 0.63), pure-tone average hearing level (musicians: 4.16 ± 1.83 dB; nonmusicians: 4.62 ± 2.61 dB; *t*_26_ = −0.54, *P* = 0.59), auditory working memory (forward and backward digit span, musicians: 12.36 ± 2.20, nonmusicians: 11.86 ± 2.28, *t*_26_ = 0.59, *P* = 0.56), and nonverbal IQ (musicians: 30.64 ± 3.89, nonmusicians: 29.28 ± 4.55, *t*_26_ = 0.85, *P* = 0.40). Musicians had started training before age 7 (mean = 5.21 ± 1.62), had at least 10 years of musical training (mean = 14.64 ± 3.46), reported practicing consistently (≥3 times per week) over the past 3 years but varied in the type of training (see Supplementary Table S1 for musicians’ details). Nonmusicians reported less than 1 year of lifetime musical experience, which did not occur in the year before the experiment.

### SIN Perception Task

This task was carried out during a previous fMRI scanning (for details, see Du and Zatorre 2017). Four 500-ms consonant-vowel syllables (/ba/, /ma/, /da/, and /ta/) with fixed 85 dB sound pressure level were randomly presented with a simultaneous 500-ms white noise segment at one of the 5 signal-to-noise ratios (SNRs: −12, −8, −4, 0, and 8 dB). Participants were required to identify syllables by pressing corresponding keys. The mean accuracy across syllables and SNRs was calculated for each individual and used as an index of SIN perception performance. As previously reported, musicians showed higher accuracy than nonmusicians in the SIN task (musicians: 76.64 ± 3.68%, nonmusicians: 68.29 ± 5.56%, *t*_26_ = 4.69, *P* < 0.001, Cohen’s *d* = 1.80). No significant correlation was found between SIN accuracy and musical training years in musicians (*r* = 0.21, *P* = 0.48). The lack of correlation may be due to a narrow range of training duration (11 ~ 22 years) without recruiting amateur or life-long musicians, as well as the small sample size.

### DTI Data Acquisition and Preprocessing

Imaging data were collected using a 3.0 T MRI system (Simens Magnetom Trio) with a 32-channel head coil. *T*_1_-weighted images were acquired using a magnetization-prepared rapid acquisition gradient echo sequence with the following parameters: repetition time (TR) = 2300 ms, echo time (TE) = 2.98 ms, flip angle = 9°, field of view (FOV) = 256 × 256 mm^2^, slice numbers = 192 slices, voxel size = 1 × 1 × 1 mm^3^. DTI data were acquired with the following parameters: diffusion-weighted gradient directions = 99, *b*-value = 1000 s/mm^2^, b0 nonweighted images = 10, TR = 9340 ms, TE = 88 ms, flip angle = 90°, FOV = 256 × 256 mm^2^, EPI factor = 128, slice numbers = 72, voxel size = 2 × 2 × 2 mm^3^. See Du and Zatorre (2017) for fMRI acquisition parameters and data analysis.

PANDA, the fully automated brain diffusion images processing software (Cui et al. 2013, http://www.nitrc.org/projects/panda/), was employed to process the raw DTI data. The core commands for preprocessing procedure were embedded in the Functional MRI of the Brain (FMRIB) software library (FSL v5.0, Smith et al. 2004, https://fsl.fmrib.ox.ac.uk/fsl/fslwiki). Firstly, the Brain Extraction Toolbox was executed on the diffusion tensor images to obtain skull stripped images. Then the diffusion images were registered to the b0 images with an affine transformation for correcting the eddy current distortion. After that, DTIFIT was applied to build tensor models, and 3 eigenvalues, λ_1,_ λ_2_, and λ_3_, were determined through tensor fitting. The λ_1_ and the averaged λ_2_ and λ_3_ were used to profile water diffusion parallel to the axonal direction (axial diffusivity, AD) and in the perpendicular direction (radial diffusivity, RD) that differentiate axon and myelin changes, respectively (Song et al. 2003). The relative ratio of AD to RD was defined as FA, which is considered as a global measure to reflect the degree of myelination and properties of axons (Song et al. 2003) and an index of microstructural ordering and integrity of fibers (Catani et al. 2007). Mean diffusivity (the average of 3 eigenvalues) was not used here since its meaning is hard to interpret. Compared with FA alone, various diffusivity measurements including FA, AD, and RD enable a better understanding of white matter properties (e.g., axons diameters, myelination) due to axonal sprouting, pruning or rerouting (Walhovd et al. 2014; Zatorre et al. 2012). The diffusivity images in individual space were then registered to a standardized template in MNI space with a voxel size of 2 × 2 × 2 mm^3^.

### Fiber Tractography

Deterministic tractography was performed to attain the probability maps of bilateral AF in each participant group using Diffusion Toolkit software (http://trackvis.org./dtk/) based on FACT tracking algorithm (Mori et al. 1999). The 3 cortical termination territories for reconstructing the tractography of AF and its 3 segments were defined according to Catani et al. (2005). The direct AF connects posterior inferior frontal gyrus (IFG) and precentral gyrus (Broca’s territory) with posterior temporal lobe (Wernicke’s territory); the anterior AF connects posterior IFG and precentral gyrus (Broca’s territory) with inferior parietal cortex (Geschwind’s territory); the posterior AF links inferior parietal cortex (Geschwind’s territory) with posterior temporal lobe (Wernicke’s territory) (Catani and Mesulam 2008). Here, using the Automated Anatomical Labeling (AAL) atlas (Tzourio-Mazoyer et al. 2002), the Broca’s territory included pars opercularis (BA44) of IFG and ventral precentral gyrus, the Geschwind’s territory included angular gyrus and supramarginal gyrus, the Wernicke’s territory contained posterior STG and middle temporal gyrus. In spite of several ways to delineate the branches of AF/SLF (Wakana et al. 2007; Catani and Mesulam 2008; Glasser and Rilling 2008), the well-studied Catani’s definition of AF segments has achieved consistency across subjects and fiber tracking methods in previous studies (Catani et al. 2007; Chen et al. 2018; Oechslin et al. 2010; Vaquero et al. 2018, 2020; Yeatman et al. 2012). As a control tract, the CST was dissected using the protocol of Wakana et al. (2007), in which the inferior termination territory was placed on the cerebral peduncle at the level of the decussation of the superior cerebellar peduncle while the superior territory was identified to cover motor fibers penetrating primary motor cortex and central sulcus.

**Figure 1:**
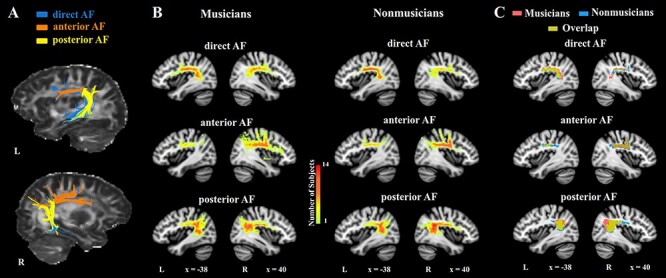
The tractography and probability maps of bilateral AF segments. (*A*) The tractography of AF segments in bilateral hemispheres in a typical individual. (*B*) The probability maps of AF segments in musicians and nonmusicians, respectively. The color represents the number of subjects in each voxel with fibers passing through. (*C*) The thresholded (≥28.6%) group probability maps of AF segments for musicians (red) and nonmusicians (blue) as well as the overlapped regions (yellow).

Before formal fiber tracking, all termination regions in MNI space were mapped into individual native space. Fiber tracking initiated from 10 randomly selected seeds within each voxel of the termination regions in the native diffusion space and terminated if the angle between 2 consecutive orientations exceeded 45° or if the FA value was lower than 0.2. It is suggested in the literature that it was impossible to reconstruct a continuous trajectory of the right direct AF in nearly a half of participants using deterministic tracking (Catani et al. 2007); for instance, the right direct AF was identified in 20 of 39 subjects in Yeatman et al. (2012), 16 of 33 and 54 of 100 subjects in Bain et al. (2019) and 32 of 54 subjects in Chen et al. (2018). Consistent with these previous findings, the right direct AF was reconstructed in 8 of 14 musicians and 9 of 14 nonmusicians here. In comparison, the left direct AF and the right anterior AF were identified in 13 musicians and 13 nonmusicians, the left anterior AF was found in 10 musicians and 11 nonmusicians, and bilateral posterior AF were reconstructed in all participants (see Table 1). Tract detection rates for the 3 segments of bilateral AF were not statistically different between groups (*P* > 0.05, Pearson’s chi-squared tests). The fiber trajectories of the 3 segments of bilateral AF in a typical individual are shown in Figure 1*A* and that of the CST are shown in Supplementary Figure S1*A*.

**Table 1:** Group comparisons of tract diffusivity measures between musicians and nonmusicians

	FA			AD (10^−3^)			RD (10^−3^)		
	Musicians M (SD)	Nonmusicians M (SD)	*P*	Musicians M (SD)	Nonmusicians M (SD)	*P*	Musicians M (SD)	Nonmusicians M (SD)	*P*
**L direct AF (13/13)**	0.47 (0.022)	0.46 (0.019)	0.148	1.13 (0.040)	1.13 (0.021)	0.913	0.52 (0.019)	0.54 (0.024)	0.030
**R direct AF (8/9)**	0.50 (0.020)	0.47 (0.025)	**0.004** ^*^	1.12 (0.029)	1.07 (0.030)	0.0002^*^	0.49 (0.024)	0.51 (0.022)	0.187
**L anterior AF (10/11)**	0.47(0.030)	0.45 (0.024)	0.021	1.09 (0.042)	1.07 (0.033)	0.319	0.50(0.021)	0.52 (0.023)	0.003^*^
**R anterior AF (13/13)**	0.43(0.024)	0.42 (0.022)	0.134	1.05 (0.026)	1.06 (0.019)	0.668	0.53(0.026)	0.55 (0.023)	0.049
**L posterior AF (14/14)**	0.41(0.015)	0.40 (0.016)	0.334	1.08 (0.029)	1.08 (0.024)	0.598	0.57(0.022)	0.58 (0.019)	0.204
**R posterior AF (14/14)**	0.36 (0.015)	0.38 (0.013)	0.020	1.04 (0.025)	1.05 (0.016)	0.326	0.59 (0.017)	0.58 (0.017)	0.211
**L CST (14/14)**	0.54 (0.016)	0.53 (0.012)	0.240	1.18 (0.032)	1.19 (0.015)	0.313	0.47 (0.024)	0.49 (0.021)	0.025
**R CST (14/14)**	0.51 (0.021)	0.50 (0.015)	0.174	1.04 (0.023)	1.04 (0.017)	0.418	0.48 (0.038)	0.49 (0.020)	0.120

Note: The numbers of participants with identified AF segment or CST are indicated in italic for musicians and nonmusicians, respectively.

*P* was estimated by permutation tests. ^*^FDR-corrected *P* < 0.05. SD, standard deviation.

Each tract trajectory was then registered to the MNI space resulting in a binary map. The overlapped binary maps in all participants of each group were used to generate a probabilistic map. The value of each voxel in the probabilistic map means the number of subjects who had fiber trajectories in that voxel. A group-level threshold was then set at voxel value >28% of subjects (4 of 14 subjects) and cluster size >240 voxels (1920 mm^3^). This approach balances the need to minimize extraneous fibers, register subtle differences of AF morphology between groups and hemispheres, and enable the correlational analysis with the behavioral variable across all subjects. Many studies on AF/SLF and other language or music-related tracts used a similar threshold as we did (25–30%, Fang et al. 2015, Oechslin et al. 2018) or even lower (10%, Loui et al. 2011). Figure 1*B* shows the probabilistic maps of AF segments in 2 groups separately, whereas that of the CST are shown in Supplementary Figure S1*B*. The overlapped and unique parts of the thresholded group-level probability maps for extracting diffusivity measures are displayed for AF segments in Figure 1*C* and the CST in Supplementary Figure S1*C*. Total voxel numbers of each tract in 2 groups are shown in Supplementary Table S2.

Lastly, for each individual, the mean diffusivity values (FA, AD, and RD) of each tract were extracted from the diffusivity maps that were overlaid with the reconstructed tract templates of each group in MNI space. The FA-based LI was then calculated for characterizing the hemispherical asymmetry of each AF segment and the CST using the following algorithm: LI = (left_FA_ − right_FA_) / (left_FA_ + right_FA_).

### Statistical Analysis

The group differences of age, education years, pure-tone hearing level, auditory digit span, nonverbal IQ, and SIN perception accuracy were examined using parametric tests (independent samples *t*-test for 2 groups). To obtain robust estimations on small samples without making any assumptions about the sampling distribution, nonparametric permutation tests were conducted on tract diffusivity or LI. Firstly, the diffusivity (FA, AD, and RD) and LI values of each tract were compared between groups by 2-sample t tests to verify the musical experience-dependent plasticity on white matter microstructure. The significance of laterality of each AF segment was additionally tested by a 1-sample *t*-test in each group separately. Next, for specifying white matter tracts involved in SIN perception, partial correlations were conducted between the diffusivity or LI values of each tract and the SIN performance, with hearing level, working memory (digit span) and nonverbal IQ as covariates. Additionally, Pearson’s correlations were performed to assess the relationships between musical training time and diffusivity or LI values. Following each above mentioned analysis, a null distribution of test statistics (*t*- or *r*-values) was generated by randomizing the labels of diffusivity or LI values 10 000 times. The permutation test *P* value was calculated by ranking of a true statistical value in the shuffled distribution ($P=\frac{ranking+1}{10\kern0.5em 000+1}$). Multiple comparisons were corrected with a FDR-corrected *q* < 0.05 using Benjamini–Hochberg procedure on 24 measurements (FA, AD, and RD of 8 tracts) or 28 measurements (FA, AD, and RD of 8 tracts plus 4 LIs).

### Mediation Analysis

To further investigate how musical experience-related white matter plasticity influenced the SIN performance, mediation analysis was conducted in AMOS software (Version 7.0) using maximum likelihood estimation to assess the direct and indirect relationships of the following variates:1) the FA value of the right direct AF that showed significant group difference and correlation with SIN performance; 2) the BOLD activity in the right STG cluster (peak Talairach coordinates: 47, −28, −1, BA 22/21, 324 mm^3^) that was significantly higher in musicians than nonmusicians during the SIN task (family-wise error corrected *P* < 0.001 by main effect of group) and predicted SIN performance in musicians (*r* = 0.53, *P* = 0.043, *N* = 15 by Pearson’s correlation) in our previous fMRI study (Du and Zatorre 2017); and 3) the SIN perception accuracy. Based on our hypothesis, a mediation model was tested with the hearing level, digit span, and nonverbal IQ as covariates: the FA of the right direct AF impacted on SIN performance via the right STG BOLD activity as a mediator (diffusivity $\rightarrow$ BOLD $\rightarrow$ SIN). The reverse model that white matter diffusivity mediated the effect of BOLD activity on SIN behavior (BOLD $\rightarrow$ diffusivity $\rightarrow$ SIN) was also tested as a supplementary verification. The bias-corrected bootstrapping method with 5000 iterations was used to estimate the 95% confidence intervals (CI). The indices of model fitting included Chi-Square statistic (χ2), its degrees of freedom and *P* value, root mean square error of approximation (RMSEA), normed-fit index (NFI), and comparative fit index (CFI). *P* of χ2 > .05, RMSEA < 0.07, NFI > 0.95, and CFI > 0.95 indicate an acceptable fit of the model (Hopper et al. 2008). It is worth mentioning that although the mediation analysis cannot confirm the causal link, it provides a way to infer the causal relationships of white matter changes on SIN performance in a statistical sense (Pearl 2012).

## Results

### Group Differences in Diffusivity

As shown in Table 1, compared with nonmusicians, musicians showed significantly higher FA (*P* = 0.004, FDR-corrected, Cohen’s *d* = 1.20) and higher AD (*P* < 0.001, FDR-corrected, Cohen’s *d* = 1.68) in the right direct AF, as well as lower RD (*P* = 0.003, FDR-corrected, Cohen’s *d* = --1.26) and a tendency of higher FA (uncorrected *P* = 0.021, Cohen’s *d* = 0.92) in the left anterior AF. The increment of FA with an increment of AD and a stable RD suggests axonal property changes in the right direct AF, whereas the increment of FA accompanied by a decrement of RD and a stable AD are compatible with an enhanced degree of myelination of the left anterior AF in musicians (Song et al. 2005; Wheeler-Kingshott and Cercignani 2009; Zatorre et al. 2012). Notably, years of musical training did not correlate with diffusivity values in bilateral AF (Supplementary Table S3, FDR-corrected *P* > 0.05). With regard to the control tract, no group difference in diffusivity was identified (FDR-corrected *P* > 0.05, Table 1) and no correlation of CST diffusivity with years of training was found (FDR-corrected *P* > 0.05, Supplementary Table S3).

### Correlation Between Diffusivity and SIN Performance

**Figure 2:**
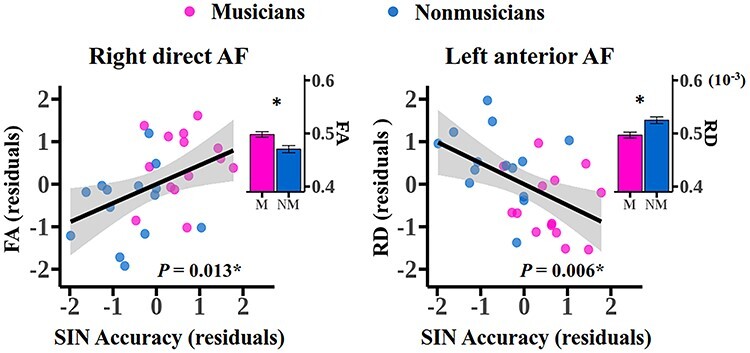
AF segments showing significant group difference and significant partial correlation with SIN perception accuracy in all participants after controlling for hearing level, digit span, and nonverbal IQ. The histograms show the group mean diffusivity and error bars represent standard errors of the mean. ^*^FDR-corrected *P* < 0.05 by permutation tests. Note that, due to the relative small sample size, the correlation estimates should be treated with caution.

To identify the white matter correlates supporting musician benefit on SIN perception in a robust way, we report only tract diffusivity values showing both a significant group difference, as well as a significant partial correlation with SIN performance after controlling for hearing level, auditory working memory and nonverbal IQ, and passing FDR correction. As shown in Figure 2, for tracts with significant group difference in diffusivity, only the FA of the right direct AF positively correlated with SIN accuracy (*P* = 0.013, FDR-corrected) and the RD of the left anterior AF negatively correlated with SIN performance (*P* = 0.006, FDR-corrected) in all participants. In addition, the RD values of the left direct AF, the right anterior AF and the left posterior AF as well as the AD values of bilateral posterior AF negatively correlated with SIN perception across the subjects (FDR-corrected *P* < 0.05 for all), although the group difference was not significant. All the correlation estimates are listed in Supplementary Table S4. Notably, the correlation estimates should be treated with caution due to the relative small sample size. When group type was additionally added as a covariate, only the AD of the left posterior AF showed a significant correlation with SIN performance (*P* = 0.001, FDR-corrected, Supplementary Table S4), which is consistent with previous finding (Vandermosten et al. 2012). As a control tract, the diffusivity of bilateral CST did not correlate with SIN accuracy (FDR-corrected *P* > 0.05 for all, Supplementary Table S4). Therefore, among a number of fibers associated with SIN processing or modulated by training, only the right direct AF and the left anterior AF were the 2 core white matter underpinnings of musician advantage in SIN perception.

### Lateralization Effect

**Figure 3:**
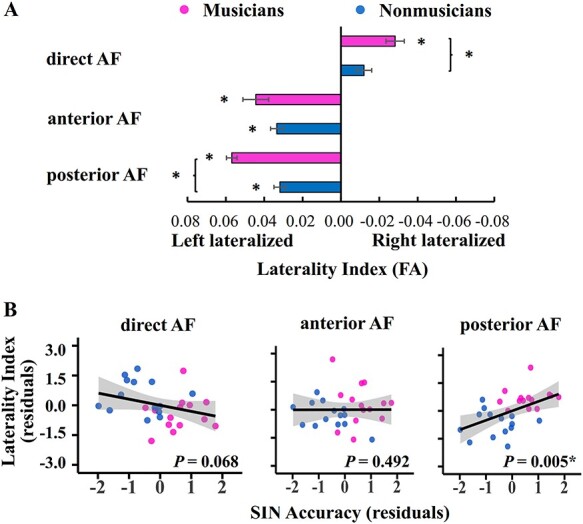
FA-based LI and its relationship with SIN performance. (*A*) LI of each AF segment in musicians and nonmusicians. Error bars indicate standard error of the mean. (*B*) Partial correlations between the LI and SIN accuracy in all participants after controlling for hearing level, digit span, nonverbal IQ. ^*^FDR-corrected *P* < 0.05 by permutation tests. Compared with nonmusicians, musicians showed stronger right lateralization of the direct AF and stronger left lateralization of the posterior AF, and stronger left lateralization of the posterior AF correlated with better SIN performance in all subjects. Note that, due to the relative small sample size, the effect size estimates should be treated with caution.

Besides looking at diffusivity of tracts in each hemisphere, laterality index [LI = (left_FA_ − right_FA_) / (left_FA_ + right_FA_)] was further calculated using FA values for characterizing each tract’s hemispheric asymmetry and its relationship with SIN performance. As shown in Figure 3*A*, in both groups the anterior AF (musicians: LI = 0.044 ± 0.025, *P* < 0.001, FDR-corrected, Cohen’s *d* = 1.77; nonmusicians: LI = 0.033 ± 0.012, *P* < 0.001, FDR-corrected, Cohen’s *d* = 2.73) and the posterior AF (musicians: LI = 0.057 ± 0.010, *P* < 0.001, FDR-corrected, Cohen’s *d* = 5.50; nonmusicians: LI = 0.032 ± 0.012, *P* < 0.001, FDR-corrected, Cohen’s *d* = 2.71) were left lateralized. The direct AF exhibited a right lateralization in musicians (LI = −0.028 ± 0.018, *P* < 0.001, FDR-corrected, Cohen’s *d* = −1.58) but no bilateral asymmetry in nonmusicians (LI = −0.012 ± 0.015, FDR-corrected *P* > 0.05). Moreover, compared with nonmusicians, musicians showed a stronger right lateralization in the direct AF (*P* = 0.016, FDR-corrected, Cohen’s *d* = −0.98) and a stronger left lateralization in the posterior AF (*P* < 0.001, FDR-corrected, Cohen’s *d* = 2.28). Among the 3 AF segments, only the LI of the posterior AF had a positive correlation with SIN performance in all participants (*P* = 0.005, FDR-corrected, Fig. 3*B*). This indicates that the musicianship-related higher degree of left lateralization of the posterior AF may serve as another neural correlate of musician advantage in SIN perception. Our result again supports previous finding that the left posterior AF contributes to speech perception in noisy environments (Vandermosten et al. 2012). No other correlation was found between the LI and SIN performance (FDR-corrected *P* > 0.05, Supplementary Table S4) or between the LI and musical training time (FDR-corrected *P* > 0.05, Supplementary Table S3).

### Mediation Analysis: Diffusivity, BOLD, and SIN Performance

**Figure 4:**
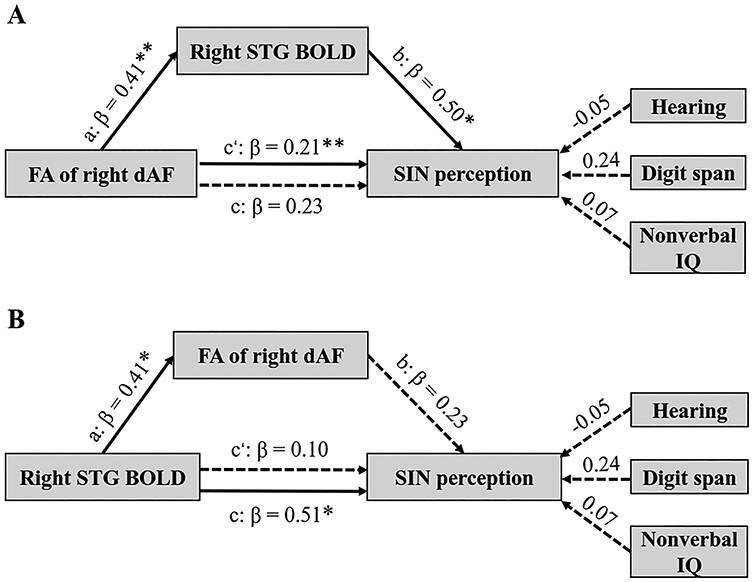
Models tested in the mediation analyses. (*A*) The FA of the right direct AF (dAF) impacted on SIN perception via the right STG BOLD activity as a mediator (diffusivity $\rightarrow$ BOLD $\rightarrow$ SIN). (*B*) The right STG BOLD activity did not influence SIN perception via the FA of the right direct AF as a mediator (BOLD $\rightarrow$ diffusivity $\rightarrow$ SIN). Dotted lines indicate insignificant paths. a, b and c mean direct pathways; c’ means indirect pathway; β means standardized path coefficient. ^*^P < 0.05, ^*^^*^P < 0.01.

As the FA of the right direct AF (*r* = 0.45, *P* = 0.013) and the BOLD activity in the right STG (*r* = 0.63, *P* < 0.001) all correlated with SIN accuracy across the entire sample of subjects, mediation analysis was performed to understand their relationships. As shown in Figure 4, Model A (diffusivity $\rightarrow$ BOLD $\rightarrow$ SIN) showed significant mediation effect and fitted the data well (χ2(9) = 9.77, *P* = 0.37; RMSEA = 0.56; NFI = 0.71; CFI = 0.96). Model A could explain 46.8% of variance in SIN perception accuracy. Specifically, the FA of the right direct AF significantly predicted the BOLD activity in the right STG (a: β = 0.41, *P* = 0.003, 95% CI = [0.14, 0.61]) which in turn significantly contributed to SIN performance (b: β = 0.50, *P* = 0.006, 95% CI = [0.20, 0.85]). Thus, the indirect effect of FA of the right direct AF on SIN performance with the right STG BOLD activity as a mediator was significant (c’: β = 0.21, *P* = 0.005, 95% CI = [0.07, 0.43]), whereas the direct effect of FA of the right direct AF on SIN accuracy was insignificant (c: β = 0.23, *P* = 0.12, 95% CI = [−0.07, 0.50]). Additionally, to test the possibility that structural changes may mediate the effect of brain function on behavioral performance, Model B with the FA of the right direct AF as a mediator in explaining the effect of the right STG BOLD activity on SIN performance (BOLD $\rightarrow$ diffusivity $\rightarrow$ SIN) was tested but failed (c’: β = 0.01, *P* = 0.076, 95% CI = [−0.12, 0.29]). These results suggest that the structural alterations of the right direct AF drove enhanced SIN perception in musicians by influencing the hemodynamic activity in the right STG.

## Discussion

In the present study, we found higher FA in the right direct AF, lower RD in the left anterior AF, and stronger left-lateralized posterior AF in musicians relative to nonmusicians; and we also found that these variables predicted better SIN perception across all participants. Thus, the microstructural organization of white matter tracts that connect auditory and frontal motor regions in both hemispheres may serve as a neural foundation of the musician advantage in understanding SIN, which supports the view that musical training strengthens sensorimotor integration in facilitating speech perception in noisy environments. Moreover, mediation analysis revealed an indirect effect for the FA of the right direct AF in predicting SIN accuracy, in which the right STG BOLD activity played a full mediation role. This finding is important in supporting a causal relationship between white matter structure, immediate brain hemodynamic function and behavior in explaining the musical training effect on speech perception.

Musical training has emerged as an efficient framework to explore brain plasticity underlying sensorimotor interactions (Herholz and Zatorre 2012). Playing music requires precisely, coherently and timely organized motor sequences, which depend on widespread anatomical and functional networks for fine-grained perception and motor control (Zatorre et al. 2007). Feedforward and feedback sensorimotor integration are implemented for monitoring, preventing and minimizing errors during music perception and production. Similarly, sensorimotor integration is involved in speech perception and production (Hickok and Poeppel 2007). Articulatory gestures based on internal models are fed forward for constraining the interpretation of acoustic patterns during speech perception, whereas acoustic features are fed back for monitoring the discrepancy between desired and actual articulation during speech production. Indeed, a unified sensorimotor integration network in speech production and speech perception was demonstrated in a recent meta-analysis of neuroimaging studies (Skipper et al. 2017). Thus, it is likely that an overlapped neuroanatomical network contributes to sensorimotor interactions in both music and speech processing, allowing a transfer effect from music domain to speech domain (Kraus and Chandrasekaran 2010).

As analysis-by-synthesis models propose, speech perception requires articulatory prediction and sensorimotor integration to promote phonetic encoding and compensate for environmental degradation (Poeppel and Monahan 2011). Accordingly, fMRI studies have demonstrated that sensorimotor integration can provide a means of compensation for decoding impoverished speech representations due to background noise or aging (Du et al. 2014; Du et al. 2016). Thus, musicians would take advantage of more efficient motor prediction and sensorimotor integration in improving speech perception in noisy circumstances. As shown in our previous fMRI study, improved SIN perception in musicians relied on stronger recruitment of auditory and frontal speech motor cortices in both hemispheres, as well as finer phonological representations in, and stronger functional connectivity between these structures (Du and Zatorre 2017). The present DTI findings support such an account that strengthened sensorimotor interaction contributes to musician advantage in SIN perception and shed light on the structural correlates underlying sensorimotor integration involved in both musical training and speech perception.

According to the dual-stream model of speech processing, the dorsal stream is involved in mapping phonological features onto articulatory representations (Hickok and Poeppel 2007). The AF is considered as the primary white matter pathway connecting posterior temporal and inferior frontal regions in support of the sensorimotor integration function of the dorsal stream. Here, musicians had significantly higher FA in the right direct AF that correlated with better SIN performance. This result is consistent with previous findings that musical training was associated with increased tract volume in the right AF (Halwani et al. 2011; Vaquero et al. 2020) and larger F1 (a directional diffusivity measure) value of the right SLF/AF (Giacosa et al. 2016). The architecture of the right AF has been related to musical abilities, such as melody and rhythm learning (Vaquero et al. 2018), pitch-related musical grammar learning (Loui et al. 2011), and it was found to be abnormal in congenital amusia (Peretz 2016; Chen et al. 2018) and acquired amusia (Sihvonen et al. 2019). In comparison, the left AF has been strongly involved in speech processing tasks. In particular, the FA of the left direct AF positively correlated with phoneme awareness (Vandermosten et al. 2012), and the mean diffusivity of the left direct AF mediated the aging effect on SIN perception (Tremblay et al. 2019).

A new finding here is the involvement of the right direct AF in SIN processing in association with musical training. This indicates a possible functional extension for stream segregation of speech sounds from background in addition to musical function of the right AF in musicians and also suggests a transition from a left lateralized dorsal pathway to a bilaterally symmetric or even right lateralized dorsal pathway as a result of musical expertise. Indeed, as revealed by the FA-based lateralization analysis, the direct AF was right lateralized in musicians but symmetric in nonmusicians. Although many studies have described a leftward laterality of the whole AF (Lebel and Beaulieu 2009) and the direct AF in particular (Catani et al. 2007) in normal population using either macro- (i.e., streamline count) or microstructural (i.e., FA) measurements, symmetric direct AF (López-Barroso et al. 2013) or right lateralized whole SLF/AF (including direct and indirect anterior tracts, Oechslin et al. 2010) have also been observed when laterality was defined by FA rather than streamlines.

A recent study using different tractography pipelines on 2 large-sample datasets found that the tractography choice and implementing the cortical constraints substantially impact the laterality measurement, which may explain the discrepant results (Bain et al. 2019). When deterministic tractography with constrained AF through defined endpoints was performed as we did here, across the 2 datasets no significant laterality of the direct AF was found if LI was calculated by voxel or streamline count, and the FA-based LI exhibited a left laterality in the anterior and posterior portions of the direct AF but a right laterality in the middle portion of the direct AF. The lack of asymmetry in nonmusicians in the present study may be partially due to diverse microstructure properties along the direct AF trajectory as well as the application of group and hemisphere-specific tract templates. Moreover, no consensus has been reached about how musical experience impacts on AF laterality. Using FA-based laterality of the group-thresholded core fibers of the whole SLF (direct plus indirect anterior AF), musicians with absolute pitch exhibited a left asymmetry, musicians with relative pitch had no asymmetry, whereas nonmusicians showed a right asymmetry (Oechslin et al. 2010). However, it is also found that musical training started at early childhood significantly reduced the leftward asymmetry in the volume of the direct AF (Vaquero et al. 2020). Interestingly, although the direct AF showed a bilateral symmetry in only 17.5% of the subjects, individuals with more symmetric direct AF were better at verbal recall (Catani et al. 2007). Thus, the better organized right AF and bilaterally symmetric or even right lateralized auditory–motor pathway may benefit speech processing in musicians.

Moreover, our previous fMRI study found that higher activity in the right STG was beneficial for SIN perception in musicians (Du and Zatorre 2017). As the mediation analysis pointed out, the FA of the right direct AF did not directly affect SIN performance but took the hemodynamic response in its terminated cortical region, the right auditory cortex, as a key mediator. It is thought that auditory cortices exhibit functional asymmetry: the left and right auditory cortices are specialized in terms of sensitivity to temporal and spectral modulation rate (Zatorre et al. 2002; Albouy et al. 2020) or in short and long temporal integration windows (Poeppel 2003), respectively. Similarly, it is reported that musicians were more sensitive to temporal (voice-onset time and duration) variations of syllables in left auditory regions and spectral (vowel) variations of syllables in right auditory regions (Kühnis et al. 2013). Presumably, a more bilaterally organized dorsal stream accompanying stronger engagement of the right auditory cortex in musicians would provide complementarity and advantage in processing speech in adverse listening environments since both temporal and spectral feature processing would be enhanced. The present finding for the first time reveals not only the white matter foundation but also its interaction with dynamic neural activity in contributing to the enhanced SIN perception in musicians.

For the posterior AF, no significant group difference was found for the diffusivity measurements. However, the FA of the posterior AF showed greater left lateralization in musicians relative to nonmusicians, and larger leftward laterality of the posterior AF as well as lower RD of the left posterior AF predicted better SIN performance. This fits well with findings that the left posterior AF contributed to phonological processing and SIN perception (Duffau 2008; Vandermosten et al. 2012). In spite of that nonmusicians generally had symmetric posterior AF in terms of the FA measurement (Catani et al. 2007; López-Barroso et al. 2013), stronger microstructural ordering and integrity of the left posterior AF relative to its right counterpart may give rise to more efficient encoding and integration of phonological representations of speech signals in musicians.

In addition, the left anterior AF showed decreased RD in musicians, which was related to enhanced SIN perception. Previous studies have shown that electrical stimulation of the anterior segment of left AF gave rise to dysarthria, suggesting its function in articulation (Duffau 2008; Maldonado et al. 2011). The musical training-related plasticity in the left anterior AF may facilitate the mapping between articulatory gestures and phonological representations in SIN perception. By examining the group difference and correlation with SIN behavior on refined AF segments in bilateral hemispheres, our findings provide insight on distinct musical experience-related plasticity in bilateral AF segments and their differential contributions to improved SIN perception.

The current study takes the first step to reveal the white matter substrates of musicians’ advantage in processing speech in adverse environments; however, some limitations exist. Firstly, the musician cohort included different types of instrumentalists as well singers, making it impossible to differentiate more detailed plasticity effects on brain structures due to the discrepancy of hands or vocal tract involvement during musical performance and their transfer effects on speech processing. Secondly, as a cross-sectional study, lacking pretraining observation of brain structures makes it difficult to distinguish between musical training-related plasticity and other environmental influences or genetic regulation on microstructural reorganization. Thirdly, the relative small sample size may lead to unstable observations on group probabilistic maps of tracts and their diffusivity, and thus reduce the reproducibility. Replications are needed with larger and more homogeneous samples in future studies. Moreover, here we focused on musicians with early age of start and relatively long-term training; amateur musicians with different training length and age of training onset should be included in future studies to explore the effects of training time and training onset on SIN perception and its structural correlates. Finally, advanced image acquisition methods like high angular resolution diffusion-weighted imaging (HARDI, Tournier et al. 2011) and analysis strategies including fiber orientation distributions function and fixel-based analysis (Raffelt et al. 2017) would solve the crossing-fiber issue, improve the tractography quality, and deepen our understanding of musicians’ advantage in processing speech.

To sum up, microstructural organization of the right direct AF and the left anterior AF, as well as stronger left lateralization of the posterior AF, may serve as structural correlates bolstering improved speech perception in noisy environments in musicians, providing new insight on the functional role of bilateral sensorimotor circuits in speech perception. In particular, mediation analysis revealed an indirect effect for the FA of the right direct AF in explaining SIN performance, in which the right STG BOLD activity played a full mediation role, highlighting the causal relationship between white matter plasticity, hemodynamic function, and behavior.

## Funding

The National Natural Science Foundation of China (grants 31671172, 31822024); the Strategic Priority Research Program of Chinese Academy of Sciences (grant XDB32010300); the Canadian Institutes of Health Research (Foundation Grant); an infrastructure grant from the Canada Fund for Innovation.

## Notes

We thank Lei Zhang, Baishen Liang, Xiaoqian Zhou, and Yiyang Wu for their help on data analysis and results examination. *Conflict of Interest*: None declared.

## Supplementary Material

Figure_S1_bhab063

Li_Zatorre_Du_Supplementary_information_bhab063

## References

[ref1] Albouy P, Benjamin L, Morillon B, Zatorre RJ. 2020. Distinct sensitivity to spectrotemporal modulation supports brain asymmetry for speech and melody. Science. 367:1043–1047. 32108113 10.1126/science.aaz3468

[ref2] Bailey JA, Zatorre RJ, Penhune VB. 2014. Early musical training is linked to gray matter structure in the ventral premotor cortex and auditory-motor rhythm synchronization performance. J Cogn Neurosci. 26:755–767. 24236696 10.1162/jocn_a_00527

[ref3] Bain JS, Yeatman JD, Schurr R, Rokem A, Mezer AA. 2019. Evaluating arcuate fasciculus laterality measurements across dataset and tractography pipelines. Hum Brain Mapp. 40:3695–3711. 31106944 10.1002/hbm.24626 PMC6679767

[ref4] Catani M, Jones DK, ffytche DH. 2005. Perisylvian language networks of the human brain. Ann Neurol. 57:8–16. 15597383 10.1002/ana.20319

[ref5] Catani M, Allin MP, Husain M, Pugliese L, Mesulam MM, Murray RM, Jones DK. 2007. Symmetries in human brain language pathways correlate with verbal recall. Proc Natl Acad Sci USA. 104:17163–17168. 17939998 10.1073/pnas.0702116104 PMC2040413

[ref6] Catani M, Mesulam M. 2008. The arcuate fasciculus and the disconnection theme in language and aphasia: history and current state. Cortex. 44:953–961. 18614162 10.1016/j.cortex.2008.04.002 PMC2740371

[ref7] Chang EF, Paygor KP, Berger MS. 2015. Contemporary model of language organization: an overview for neurosurgeons. J Neurosurg. 122:250–261. 25423277 10.3171/2014.10.JNS132647

[ref8] Chen XZ, Zhao YX, Zhong SY, Cui ZX, Li JQ, Gong GL, Dong Q, Nan Y. 2018. The lateralized arcuate fasciculus in developmental pitch disorders among mandarin amusics: left for speech and right for music. Brain Struct Funct. 223:2013–2024. 29322239 10.1007/s00429-018-1608-2

[ref9] Coffey EBJ, Mogilever NB, Zatorre RJ. 2017a. Speech-in-noise perception in musicians: a review. Hear Res. 352:49–69. 28213134 10.1016/j.heares.2017.02.006

[ref10] Coffey EBJ, Chepesiuk AMP, Herholz SC, Baillet S, Zatorre RJ. 2017b. Neural correlates of early sound encoding and their relationship to speech-in-noise perception. Front Neurosci. 11:479. 28890684 10.3389/fnins.2017.00479 PMC5575455

[ref11] Cui Z, Zhong S, Xu P, Gong G, He Y. 2013. PANDA: a pipeline toolbox for analyzing brain diffusion images. Front Hum Neurosci. 7:42. 23439846 10.3389/fnhum.2013.00042 PMC3578208

[ref12] Du Y, Buchsbaum BR, Grady CL, Alain C. 2014. Noise differentially impacts phoneme representations in the auditory and speech motor systems. Proc Natl Acad Sci USA. 111:7126–7131. 24778251 10.1073/pnas.1318738111 PMC4024897

[ref13] Du Y, Buchsbaum BR, Grady CL, Alain C. 2016. Increased activity in frontal motor cortex compensates impaired speech perception in older adults. Nat Commun. 7:12241. 27483187 10.1038/ncomms12241 PMC4974649

[ref14] Du Y, Zatorre RJ. 2017. Musical training sharpens and bonds ears and tongue to hear speech better. Proc Natl Acad Sci USA. 114:13579–13584. 29203648 10.1073/pnas.1712223114 PMC5754781

[ref15] Duffau H. 2008. The anatomo-functional connectivity of language revisited. New insights provided by electrostimulation and tractography. Neuropsychologia. 46:927–934. 18093622 10.1016/j.neuropsychologia.2007.10.025

[ref16] Fang YX, Han ZZ, Zhong SY, Gong GL, Song LP, Liu FS, Huang RW, Du XX, Sun R, Wang Q, et al. 2015. The semantic anatomical network: evidence from healthy and brain-damaged patient populations. Hum Brain Mapp. 36:3499–3515. 26059098 10.1002/hbm.22858 PMC6869673

[ref17] Frey S, Campbell JS, Pike GB, Petrides M. 2008. Dissociating the human language pathways with high angular resolution diffusion fiber tractography. J Neurosci. 28:11435–11444. 18987180 10.1523/JNEUROSCI.2388-08.2008 PMC6671318

[ref18] Giacosa C, Karpati FJ, Foster NE, Penhune VB, Hyde KL. 2016. Dance and music training have different effects on white matter diffusivity in sensorimotor pathways. Neuroimage. 135:273–286. 27114054 10.1016/j.neuroimage.2016.04.048

[ref19] Gierhan AME. 2013. Connections for auditory language in the human brain. Brain Lang. 127:205–221. 23290461 10.1016/j.bandl.2012.11.002

[ref20] Glasser MF, Rilling JK. 2008. DTI tractography of the human brain's language pathways. Cereb Cortex. 18:2471–2482. 18281301 10.1093/cercor/bhn011

[ref21] Halwani GF, Loui P, Ruber T, Schlaug G. 2011. Effects of practice and experience on the arcuate fasciculus: comparing singers, instrumentalists, and non-musicians. Front Psychol. 2:156. 21779271 10.3389/fpsyg.2011.00156 PMC3133864

[ref22] Herholz SC, Zatorre RJ. 2012. Musical training as a framework for brain plasticity: behavior, function, and structure. Neuron. 76:486–502. 23141061 10.1016/j.neuron.2012.10.011

[ref24] Hickok G, Poeppel D. 2007. The cortical organization of speech processing. Nat Rev Neurosci. 8:393–402. 17431404 10.1038/nrn2113

[ref25] Hopper D, Coughlan J, Mullen MR. 2008. Structural equation modeling: guidelines for determining model fit. Electron J Bus Res Methods. 6:53–60.

[ref26] Imfeld A, Oechslin MS, Meyer M, Loenneker T, Jancke L. 2009. White matter plasticity in the corticospinal tract of musicians: a diffusion tensor imaging study. Neuroimage. 46:600–607. 19264144 10.1016/j.neuroimage.2009.02.025

[ref27] Kraus N, Chandrasekaran B. 2010. Music training for the development of auditory skills. Nat Rev Neurosci. 11:599–605. 20648064 10.1038/nrn2882

[ref28] Kraus N, Strait DL, Parbery-Clark A. 2012. Cognitive factors shape brain networks for auditory skills: spotlight on auditory working memory. Ann N Y Acad Sci. 1252:100–107. 22524346 10.1111/j.1749-6632.2012.06463.x PMC3338202

[ref29] Kühnis J, Elmer S, Meyer M, Jäncke L. 2013. The encoding of vowels and temporal speech cues in the auditory cortex of professional musicians: an EEG study. Neuropsychologia. 51:1608–1618. 23664833 10.1016/j.neuropsychologia.2013.04.007

[ref69] Lebel C, Beaulieu C. 2009. Lateralization of the arcuate fasciculus from childhood to adulthood and its relation to cognitive abilities in children. Hum Brain Mapp. 30:3563–3573. 19365801 10.1002/hbm.20779 PMC6870654

[ref30] López-Barroso D, Catani M, Ripolles P, Dell'Acqua F, Rodríguez-Fornells A, de Diego-Balaguer R. 2013. Word learning is mediated by the left arcuate fasciculus. Proc Natl Acad Sci USA. 110:13168–13173. 23884655 10.1073/pnas.1301696110 PMC3740909

[ref31] Lu C, Qi Z, Harris A, Weil LW, Han M, Halverson K, Perrachione TK, Kjelgaard M, Wexler K, Tager-Flusberg H, et al. 2016. Shared neuroanatomical substrates of impaired phonological working memory across reading disability and autism. Biol Psychiatry Cogn Neurosci Neuroimaging. 1:169–177. 26949750 10.1016/j.bpsc.2015.11.001 PMC4776338

[ref32] Luo C, Guo ZW, Lai YX, Liao W, Liu Q, Kendrick KM, Yao DZ, Li H. 2012. Musical training induces functional plasticity in perceptual and motor networks: insights from resting-state FMRI. PLoS One. 7:e36568. 22586478 10.1371/journal.pone.0036568 PMC3346725

[ref33] Loui P, Charles Li H, Schlaug G. 2011, 2011. White matter integrity in right hemisphere predicts pitch-related grammar learning. Neuroimage. (55):500–507. 21168517 10.1016/j.neuroimage.2010.12.022 PMC3035724

[ref34] Maldonado IL, Moritz-Gasser S, Duffau H. 2011. Does the left superior longitudinal fascicle subserve language semantics? A brain electrostimulation study. Brain Struct Funct. 216:263–274. 21538022 10.1007/s00429-011-0309-x

[ref70] Martino J, De Witt Hamer PC, Berger MS, Lawton MT, Arnold CM, de Lucas EM, Duffau H. 2013. Analysis of the subcomponents and cortical terminations of the perisylvian superior longitudinal fasciculus: a fiber dissection and DTI tractography study. Brain Struct Funct. 218:105–121. 22422148 10.1007/s00429-012-0386-5

[ref35] Mori S, Crain BJ, Chacko VP, van Zijl PC. 1999. Three-dimensional tracking of axonal projections in the brain by magnetic resonance imaging. Ann Neurol. 45:265–269. 9989633 10.1002/1531-8249(199902)45:2<265::aid-ana21>3.0.co;2-3

[ref36] Oechslin MS, Imfeld A, Loenneker T, Meyer M, Jäncke L. 2010. The plasticity of the superior longitudinal fasciculus as a function of musical expertise: a diffusion tensor imaging study. Front Hum Neurosci. 3:76. 20161812 10.3389/neuro.09.076.2009 PMC2821183

[ref37] Oechslin MS, Gschwind M, James CE. 2018. Tracking training-related plasticity by combining fMRI and DTI: the right hemisphere ventral stream mediates musical syntax processing. Cereb Cortex. 28:1209–1218. 28203797 10.1093/cercor/bhx033

[ref38] Palomar-Garcia MA, Zatorre RJ, Ventura-Campos N, Bueicheku E, Avila C. 2017. Modulation of functional connectivity in auditory-motor networks in musicians compared with nonmusicians. Cereb Cortex. 27:2768–2778. 27166170 10.1093/cercor/bhw120

[ref39] Parbery-Clark A, Skoe E, Lam C, Kraus N. 2009. Musician enhancement for speech-in-noise. Ear Hear. 30:653–661. 19734788 10.1097/AUD.0b013e3181b412e9

[ref40] Parbery-Clark A, Tierney A, Strait DL, Kraus N. 2012. Musicians have fine-tuned neural distinction of speech syllables. Neuroscience. 219:111–119. 22634507 10.1016/j.neuroscience.2012.05.042 PMC3402586

[ref41] Pearl J. 2012. The causal foundations of structural equation modeling. In: Hoyle RH, editor. Handbook of structural equation modeling. New York: The Guilford Press, pp. 68–91.

[ref42] Peretz I. 2016. Neurobiology of congenital amusia. Trends Cogn Sci. 20:857–867. 27692992 10.1016/j.tics.2016.09.002

[ref43] Poeppel D. 2003. The analysis of speech in different temporal integration windows: cerebral lateralization as ‘asymmetric sampling in time. Speech Commun. 41:245–255.

[ref44] Poeppel D, Monahan PJ. 2011. Feedforward and feedback in speech perception: revisiting analysis by synthesis. Lang Cogn Process. 26:935–951.

[ref45] Puschmann S, Baillet S, Zatorre RJ. 2019. Musicians at the cocktail party: neural substrates of musical training during selective listening in multispeaker situations. Cereb Cortex. 29:3253–3265. 30137239 10.1093/cercor/bhy193 PMC6644853

[ref46] Raffelt DA, Tournier J-D, Smith RE, Vaughan DN, Jackson G, Ridgway GR, Connelly A. 2017. Investigating white matter fibre density and morphology using fixel-based analysis. Neuroimage. 144:58–73. 27639350 10.1016/j.neuroimage.2016.09.029 PMC5182031

[ref47] Saur D, Kreher BW, Schnell S, Kümmerer D, Kellmeyer P, Vry M-S, Umarova R, Musso M, Glauche V, Abel S. 2008. Ventral and dorsal pathways for language. Proc Natl Acad Sci USA. 105:18035–18040. 19004769 10.1073/pnas.0805234105 PMC2584675

[ref48] Sihvonen AJ, Särkämö T, Rodríguez-Fornells A, Ripollés P, Münte TF, Soinila S. 2019. Neural architectures of music—insights from acquired amusia. Neurosci Biobehav Rev. 107:104–114. 31479663 10.1016/j.neubiorev.2019.08.023

[ref49] Skipper JI, Devlin JT, Lametti DR. 2017. The hearing ear is always found close to the speaking tongue: review of the role of the motor system in speech perception. Brain Lang. 164:77–105. 27821280 10.1016/j.bandl.2016.10.004

[ref50] Smith SM, Jenkinson M, Woolrich MW, Beckmann CF, Behrens TE, Johansen-Berg H, Bannister PR, De Luca M, Drobnjak I, Flitney DE. 2004. Advances in functional and structural MR image analysis and implementation as FSL. Neuroimage. 23:S208–S219. 15501092 10.1016/j.neuroimage.2004.07.051

[ref51] Song S-K, Sun S-W, Ju W-K, Lin S-J, Cross AH, Neufeld AH. 2003. Diffusion tensor imaging detects and differentiates axon and myelin degeneration in mouse optic nerve after retinal ischemia. Neuroimage. 20:1714–1722. 14642481 10.1016/j.neuroimage.2003.07.005

[ref73] Song SK, Yoshino J, Le TQ, Lin SJ, Sun SW, Cross AH, Armstrong RC. 2005. Demyelination increases radial diffusivity in corpus callosum of mouse brain. Neuroimage. 26:132–140. 15862213 10.1016/j.neuroimage.2005.01.028

[ref52] Strait DL, Kraus N. 2011. Can you hear me now? Musical training shapes functional brain networks for selective auditory attention and hearing speech in noise. Front Psychol. 2:113. 21716636 10.3389/fpsyg.2011.00113 PMC3115514

[ref53] Tournier JD, Mori S, Leemans A. 2011. Diffusion tensor imaging and beyond. Magn Reson Med. 65:1532–1556. 21469191 10.1002/mrm.22924 PMC3366862

[ref54] Tremblay P, Perron M, Deschamps I, Kennedy-Higgins D, Houde JC, Dick AS, Descoteaux M. 2019. The role of the arcuate and middle longitudinal fasciculi in speech perception in noise in adulthood. Hum Brain Mapp. 40:226–241. 30277622 10.1002/hbm.24367 PMC6865648

[ref55] Tzourio-Mazoyer N, Landeau B, Papathanassiou D, Crivello F, Etard O, Delcroix N, Mazoyer B, Joliot M. 2002. Automated anatomical labeling of activations in SPM using a macroscopic anatomical parcellation of the MNI MRI single-subject brain. Neuroimage. 15:273–289. 11771995 10.1006/nimg.2001.0978

[ref56] Vandermosten M, Boets B, Poelmans H, Sunaert S, Wouters J, Ghesquiere P. 2012. A tractography study in dyslexia: neuroanatomic correlates of orthographic, phonological and speech processing. Brain. 135:935–948. 22327793 10.1093/brain/awr363

[ref57] Vaquero L, Rodríguez-Fornells A, Reiterer SM. 2017. The left, the better: white-matter brain integrity predicts foreign language imitation ability. Cereb Cortex. 27:3906–3917. 27461123 10.1093/cercor/bhw199

[ref58] Vaquero L, Ramos-Escobar N, Francois C, Penhune V, Rodriguez-Fornells A. 2018. White-matter structural connectivity predicts short-term melody and rhythm learning in non-musicians. Neuroimage. 181:252–262. 29929006 10.1016/j.neuroimage.2018.06.054

[ref59] Vaquero L, Rousseau PN, Vozian D, Klein D, Penhune V. 2020. What you learn & when you learn it: impact of early bilingual & music experience on the structural characteristics of auditory-motor pathways. Neuroimage. 213:116689. 32119984 10.1016/j.neuroimage.2020.116689

[ref60] Wakana S, Caprihan A, Panzenboeck MM, Fallon JH, Perry M, Gollub RL, Hua K, Zhang J, Jiang H, Dubey P, et al. 2007. Reproducibility of quantitative tractography methods applied to cerebral white matter. Neuroimage. 36:630–644. 17481925 10.1016/j.neuroimage.2007.02.049 PMC2350213

[ref71] Walhovd KB, Johansen-Berg H, Karadottir RT. 2014. Unraveling the secrets of white matter--bridging the gap between cellular, animal and human imaging studies. Neuroscience. 276:2–13. 25003711 10.1016/j.neuroscience.2014.06.058 PMC4155933

[ref61] Wang X, Pathak S, Stefaneanu L, Yeh FC, Li S, Fernandez-Miranda JC. 2016. Subcomponents and connectivity of the superior longitudinal fasciculus in the human brain. Brain Struct Funct. 221:2075–2092. 25782434 10.1007/s00429-015-1028-5

[ref72] Wheeler-Kingshott CA, Cercignani M. 2009. About ``axial'' and ``radial'' diffusivities. Magn Reson Med. 61:1255–1260. 19253405 10.1002/mrm.21965

[ref62] Yeatman JD, Dougherty RF, Ben-Shachar M, Wandell BA. 2012. Development of white matter and reading skills. Proc Natl Acad Sci USA. 109:E3045–E3053. 23045658 10.1073/pnas.1206792109 PMC3497768

[ref63] Yoo J, Bidelman GM. 2019. Linguistic, perceptual, and cognitive factors underlying musicians’ benefits in noise-degraded speech perception. Hear Res. 377:189–195. 30978607 10.1016/j.heares.2019.03.021 PMC6511496

[ref64] Zamorano AM, Cifre I, Montoya P, Riquelme I, Kleber B. 2017. Insula-based networks in professional musicians: evidence for increased functional connectivity during resting state fMRI. Hum Brain Mapp. 38:4834–4849. 28737256 10.1002/hbm.23682 PMC6866802

[ref65] Zatorre RJ, Belin P, Penhune VB. 2002. Structure and function of auditory cortex: music and speech. Trends Cogn Sci. 6:37–46. 11849614 10.1016/s1364-6613(00)01816-7

[ref66] Zatorre RJ, Chen JL, Penhune VB. 2007. When the brain plays music: auditory-motor interactions in music perception and production. Nat Rev Neurosci. 8:547–558. 17585307 10.1038/nrn2152

[ref74] Zatorre RJ, Fields RD, Johansen-Berg H. 2012. Plasticity in gray and white: neuroimaging changes in brain structure during learning. Nat Neurosci. 15:528–536. 22426254 10.1038/nn.3045 PMC3660656

[ref67] Zendel BR, West GL, Belleville S, Peretz I. 2019. Musical training improves the ability to understand speech-in-noise in older adults. Neurobiol Aging. 81:102–115. 31280114 10.1016/j.neurobiolaging.2019.05.015

[ref68] Zhang L, Fu X, Luo D, Xing L, Du Y. 2021. Musical experience offsets age-related decline in understanding speech-in-noise: type of training does not matter, working memory is the key.Ear Hear. 42:258–270. 10.1097/AUD.0000000000000921 PMC7969154 32826504

