# Supplementary figures and images for: The Microstructural Plasticity of the Arcuate Fasciculus Undergirds Improved Speech in Noise Perception in Musicians
Source: Cereb Cortex. 2021 May 26;31(9):3975–85. doi: 10.1093/cercor/bhab063 (PMC8328222; doi:10.1093/cercor/bhab063)

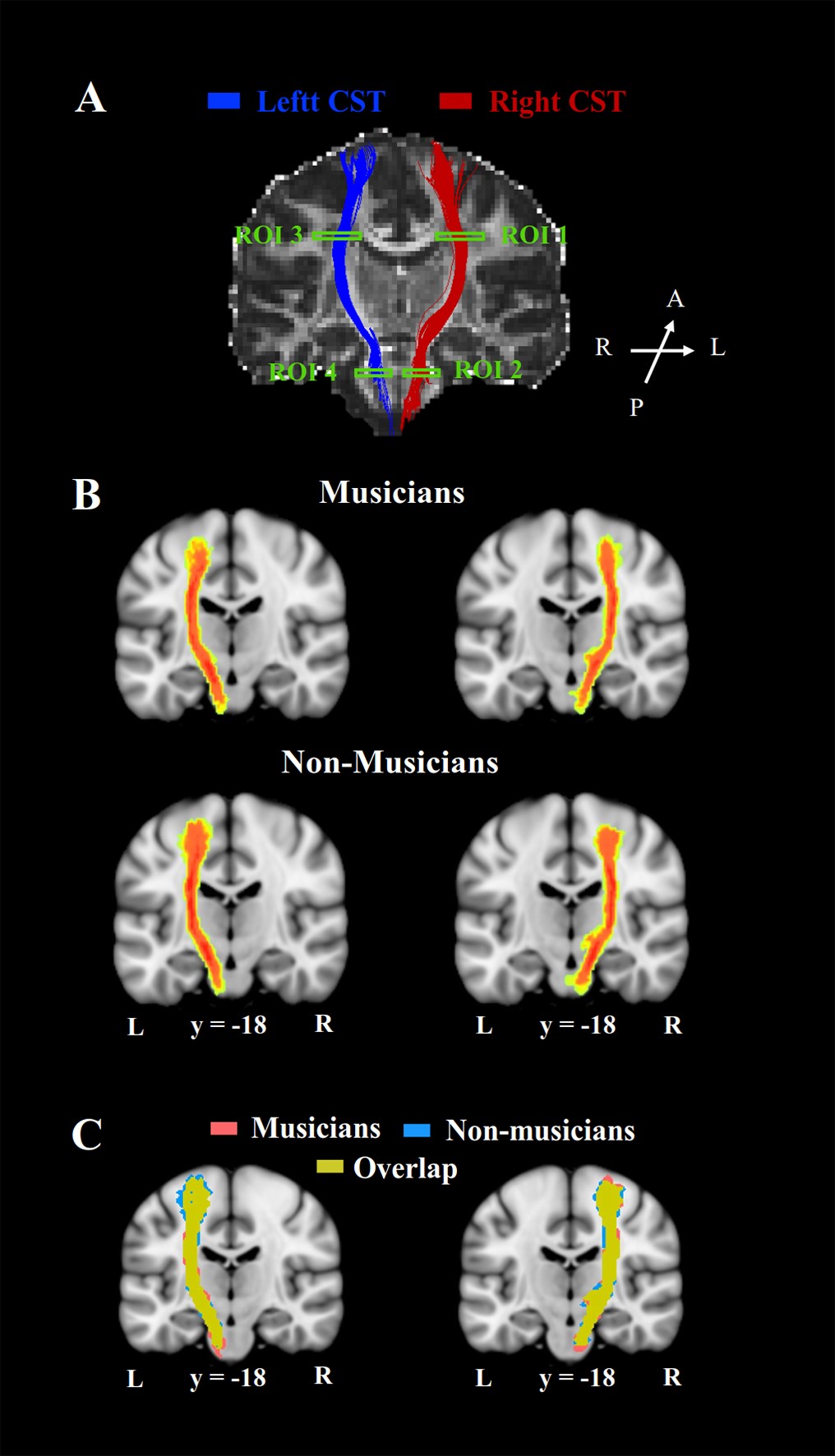

Supplement: Figure_S1_bhab063 [file figure_s1_bhab063.jpeg]
