# Supplementary material for: The Microstructural Plasticity of the Arcuate Fasciculus Undergirds Improved Speech in Noise Perception in Musicians
Source: Cereb Cortex. 2021 May 26;31(9):3975–85. doi: 10.1093/cercor/bhab063 (PMC8328222; doi:10.1093/cercor/bhab063)
Supplement: Li_Zatorre_Du_Supplementary_information_bhab063 [file li_zatorre_du_supplementary_information_bhab063.docx]

**Supplementary information**

**Table S1.** Details of musicians’ musical experience.

| **Participant ID** | **Years of**  **training** | **Age of**  **training onset** | **Type of training** |
| --- | --- | --- | --- |
| 1 | 18 | 5 | Piano, flute, guitar |
| 2 | 13 | 7 | Voice, electric bass, guitar |
| 3 | 12 | 7 | Flute, piano, voice |
| 4 | 12 | 7 | Saxophone, piano, voice |
| 5 | 15 | 4 | Guitar, drum, piano, voice |
| 6 | 14 | 7 | Voice |
| 7 | 11 | 7 | Double bass, piano |
| 8 | 22 | 3 | Oboe, piano |
| 9 | 16 | 5 | Piano, harpsichord |
| 10 | 17 | 6 | Clarinet |
| 11 | 18 | 5 | Bassoon, piano, flute |
| 12 | 19 | 3 | Piano, trumpet, flute, voice |
| 13 | 18 | 3 | Voice, piano, clarinet, trumpet |
| 14 | 16 | 4 | Trumpet, piano, |

**Table S2.** The number of voxels in the thresholded probability maps of AF segments and the CST.

| **Tract** | **Musicians** | **Non-musicians** |
| --- | --- | --- |
| L direct AF | 680 | 609 |
| R direct AF | 269 | 246 |
| L anterior AF | 233 | 248 |
| R anterior AF | 805 | 837 |
| L posterior AF | 936 | 937 |
| R posterior AF | 1306 | 1274 |
| L corticospinal | 2183 | 2190 |
| R corticospinal | 2262 | 2461 |

**Table S3.** Pearson’s correlations between years of musical training and tract diffusivity and laterality index.

| **Tract** | **FA** | **AD** | **RD** | **LI** |
| --- | --- | --- | --- | --- |
| **L direct AF** | -0.71 (0.002) | -0.45 (0.049) | 0.52 (0.032) | -0.26 (0.182) |
| **R direct AF** | -0.60 (0.014) | -0.23 (0.216) | 0.42 (0.067) |  |
| **L anterior AF** | **-**0.62 (0.010) | -0.37 (0.095) | 0.59 (0.014) | -0.27 (0.182) |
| **R anterior AF** | -0.47 (0.043) | -0.28 (0.162) | 0.35 (0.109) |  |
| **L posterior AF** | -0.38 (0.100) | -0.36 (0.099) | 0.08 (0.377) | 0.55 (0.026) |
| **R posterior AF** | -0.59 (0.014) | -0.38 (0.083) | 0.29 (0.157) |  |
| **L CST** | -0.42 (0.068) | -0.006 (0.480) | 0.35 (0.105) | 0.05 (0.431) |
| **R CST** | -0.33 (0.123) | -0.27 (0.174) | 0.20 (0.254) |  |

AF, arcuate fasciculus; CST, corticospinal tract; FA, fractional anisotropy; AD, axial diffusivity; RD, radial diffusivity; LI, laterality index. Values are *r* (*P*); *P* was estimated based on permutation tests; None passed FDR correction.

**Table S4.** Partial correlations between speech-in-noise perception accuracy and tract diffusivity and laterality measures across all the participants after controlling for hearing level, digit span, non-verbal IQ and group type.

|  | **Group type not controlled** | | | | **Group type controlled** | | | |
| --- | --- | --- | --- | --- | --- | --- | --- | --- |
|  | **FA** | **AD** | **RD** | **LI** | **FA** | **AD** | **RD** | **LI** |
| **L direct AF** | 0.31  (0.068) | -0.28  (0.087) | **-0.56 (0.002)*** | -0.31 (0.068) | 0.17 (0.212) | -0.37 (0.038) | -0.44 (0.017) | 0.01 (0.477) |
| **R direct AF** | **0.45 (0.013)*** | 0.31  (0.064) | -0.39  (0.024) |  | 0.15 (0.236) | -0.30 (0.076) | -0.32 (0.067) |  |
| **L anterior AF** | 0.28  (0.083) | -0.07  (0.370) | **-0.50 (0.006)*** | -0.003 (0.492) | -0.01 (0.465) | -0.30 (0.08) | -0.22 (0.158) | -0.29 (0.084) |
| **R anterior AF** | 0.31  (0.061) | -0.21  (0.157) | **-0.45 (0.012)*** |  | 0.17 (0.207) | -0.22 (0.150) | -0.30 (0.080) |  |
| **L posterior AF** | 0.25  (0.113) | **-0.49 (0.006)*** | **-0.49 (0.007)*** | **0.51 (0.005)*** | 0.15 (0.238) | **-0.60 (0.001)*** | -0.45 (0.013) | 0.01 (0.490) |
| **R posterior AF** | -0.17  (0.201) | **-0.51 (0.006)*** | -0.13  (0.263) |  | 0.15 (0.240) | -0.52 (0.006) | -0.40 (0.030) |  |
| **L CST** | 0.21  (0.156) | -0.10  (0.315) | -0.32  (0.060) | -0.05 (0.392) | 0.06 (0.386) | 0.07 (0.382) | -0.04 (0.435) | 0.04 (0.415) |
| **R CST** | 0.18  (0.186) | -0.38  (0.031) | -0.28  (0.082) |  | 0.02 (0.465) | -0.38 (0.033) | -0.10 (0.327) |  |

AF, arcuate fasciculus; CST, corticospinal tract; FA, fractional anisotropy; AD, axial diffusivity; RD, radial diffusivity; LI, laterality index. Values are *r (P)*. *P* was estimated by permutation tests. * FDR-corrected *P* < 0.05. Note that, due to the relative small sample size, the correlation estimates should be treated with caution.

**Supplementary figure legends**

**Figure S1.** The tractography and probability maps of bilateral corticospinal tracts (CST). (**A**) The tractography of the CST in a typical individual. The upper and lower green rectangles in each hemisphere indicate superior and inferior regions of interest (ROI) to constrain bilateral CST. (**B**) The probability maps of the CST in musicians and non-musicians, respectively. The color represents the number of subjects in each voxel with fibers passing through. (**C**) The thresholded (> 28%) group probability maps of the CST for musicians (red) and non-musicians (blue) as well as the overlapped regions (yellow).
